# 405. Searching for the Path of Least Resistance: Characterizing Longitudinal Changes in the Gut Resistomes of Healthy Infants and NICU Infants

**DOI:** 10.1093/ofid/ofaf695.143

**Published:** 2026-01-11

**Authors:** Rachel Strength, Emily Robbins, Shira Levy, Angelina G Angelova, Kathryn E McCauley, Ruhika Prasad, Phoebe LaPoint, Mickayla Bacorn, Hector Romero Soto, Kevin Lloyd, Aimee Dassner, Esther Esadah, Rana F Hamdy, Craig A Shapiro, Joseph M Campos, Suchitra Hourigan

**Affiliations:** NIH/NIAID/Children's National Hospital, SIlver Spring, MD; NIH, Bethesda, Maryland; NIAID/NIH, Wheaton, Maryland; NIH, Bethesda, Maryland; National Institutes of Allergy and Infectious Diseases, Bethesda, Maryland; NIAID/NIH, Wheaton, Maryland; NIAID, NIH, Bethesda, Maryland; NIAID, NIH, Bethesda, Maryland; National Institutes of Health, Silver Spring, Maryland; Childrens National Hospital, Washington, District of Columbia; Children's National Health System, Washington, District of Columbia; Children's National Hospital, Washington, District of Columbia; Childrens National Hospital, Washington, District of Columbia; Children's National Hospital, Washington, District of Columbia; Children's National Hospital, Washington, District of Columbia; NIAID, NIH, Bethesda, Maryland

## Abstract

**Background:**

214,000 infants die annually from antimicrobial resistance worldwide. Clinically-important antibiotic resistance genes (CI-ARG) drive these poor outcomes and impact antibiotic selection. The infant gut harbors many CI-ARG, and the pool of all gut resistance genes comprises the gut resistome. While antibiotics have been shown to increase CI-ARG abundance in older patients, longitudinal CI-ARG development of infants with neonatal intensive care unit (NICU) exposure is not well characterized.

**Figure:**
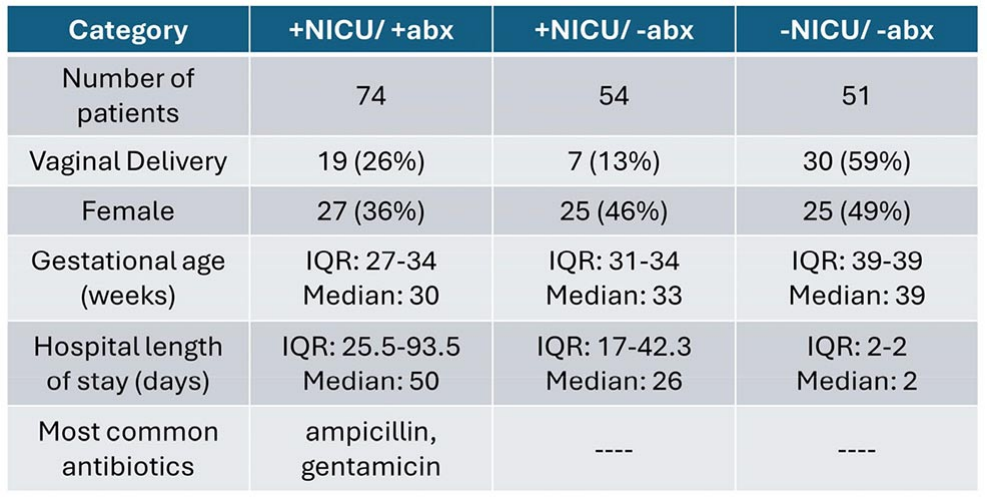
Patient Demographics The three study groups are quite different from one another in terms of vaginal deliveries, gestational age, and hospital length of stay. IQR = interquartile range.

**Figure:**
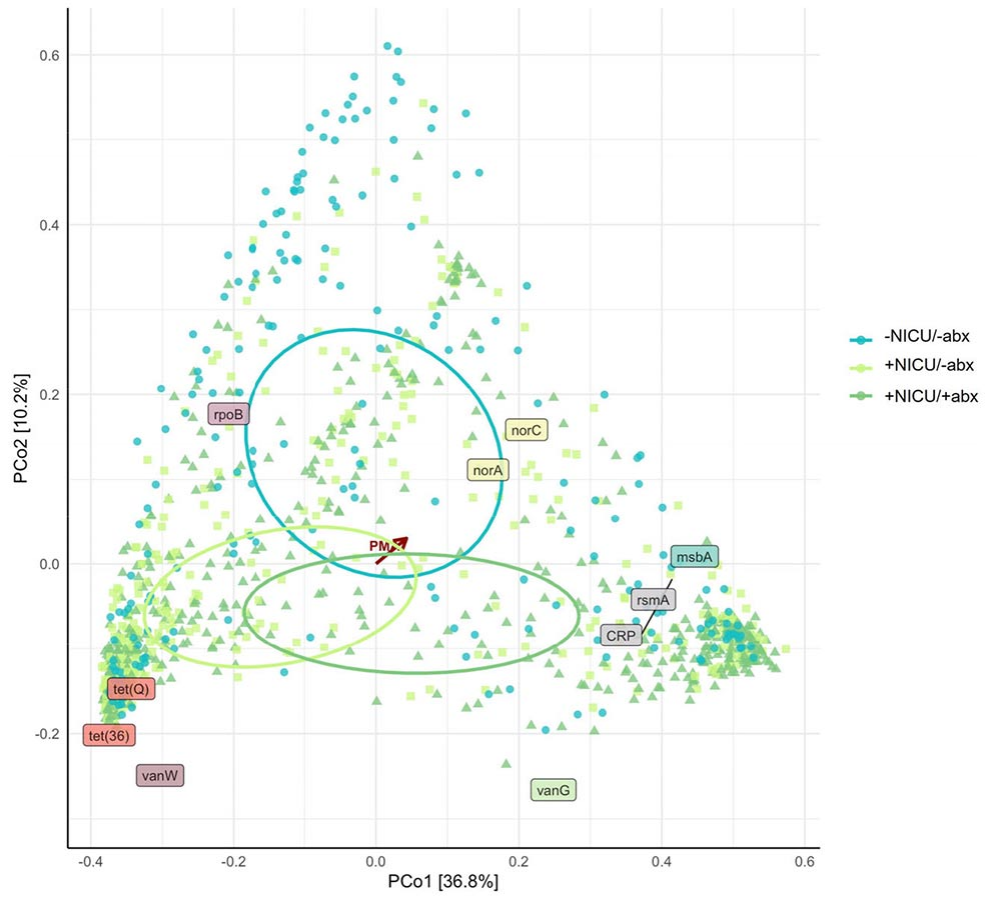
Principal Coordinates Analysis Plot of Resistome Beta Diversity with Bray-Curtis Dissimilarity Distances Beta diversity of ARG was significantly different between the three study groups over time, even after adjustment for inter-personal variation (p = 0.003). The resistance genes that drive these differences are noted. Older postmenstrual age is correlated with movement of sample resistome composition in the direction of the red arrow.

**Methods:**

We compared CI-ARG longitudinally up to 3 years of age in 3 groups, categorized by initial NICU admission and antibiotic (abx) exposure: +NICU/+abx (74 infants), +NICU/-abx (54 infants), -NICU/-abx (51 infants) (Figure 1). Metagenomic shotgun sequencing was performed on 1141 serial stool samples. To account for inter-group gestational age differences, linear mixed effects models were used to analyze CI-ARG abundance over postmenstrual age. CI-ARG included specific genes like *vanB* as well as groups of genes conferring resistance to antimicrobials such as carbapenems and cephalosporins. We also collected clinical metadata such as mode of delivery, breastfeeding, and later antibiotic use.

Hypothesis: The +NICU/+abx group will have more abundant CI-ARG longitudinally than the +NICU/-abx or -NICU/-abx groups.

**Figure:**
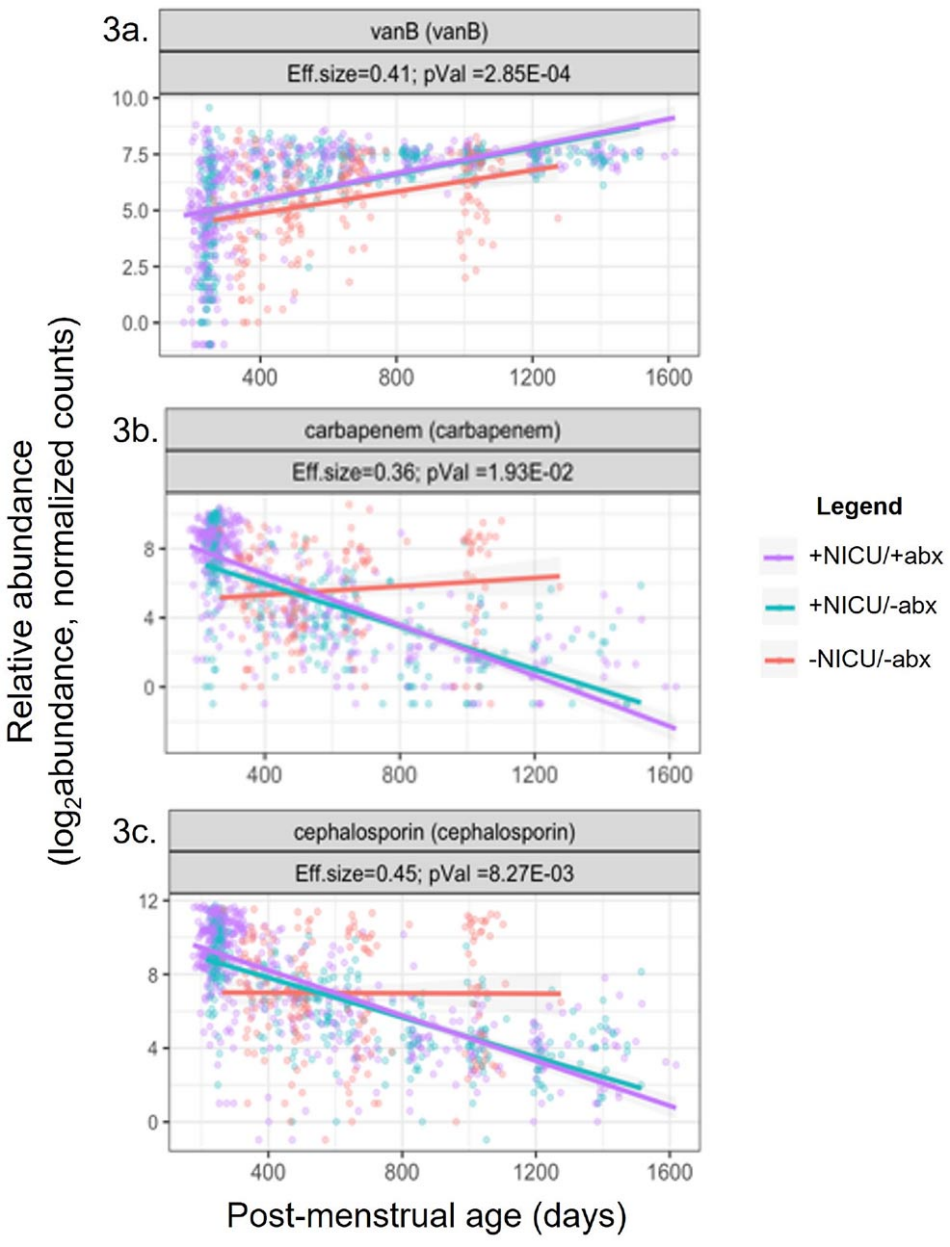
Linear Mixed Effects Models of Relative CI-ARG Abundance versus Postmenstrual Age Each dot in the plots shows relative abundance and postmenstrual age of each sample for 3 representative CI-ARG: vanB, carbapenems, and cephalosporins. Linear mixed effects models were then applied to assess trends in the data.

**Figure:**
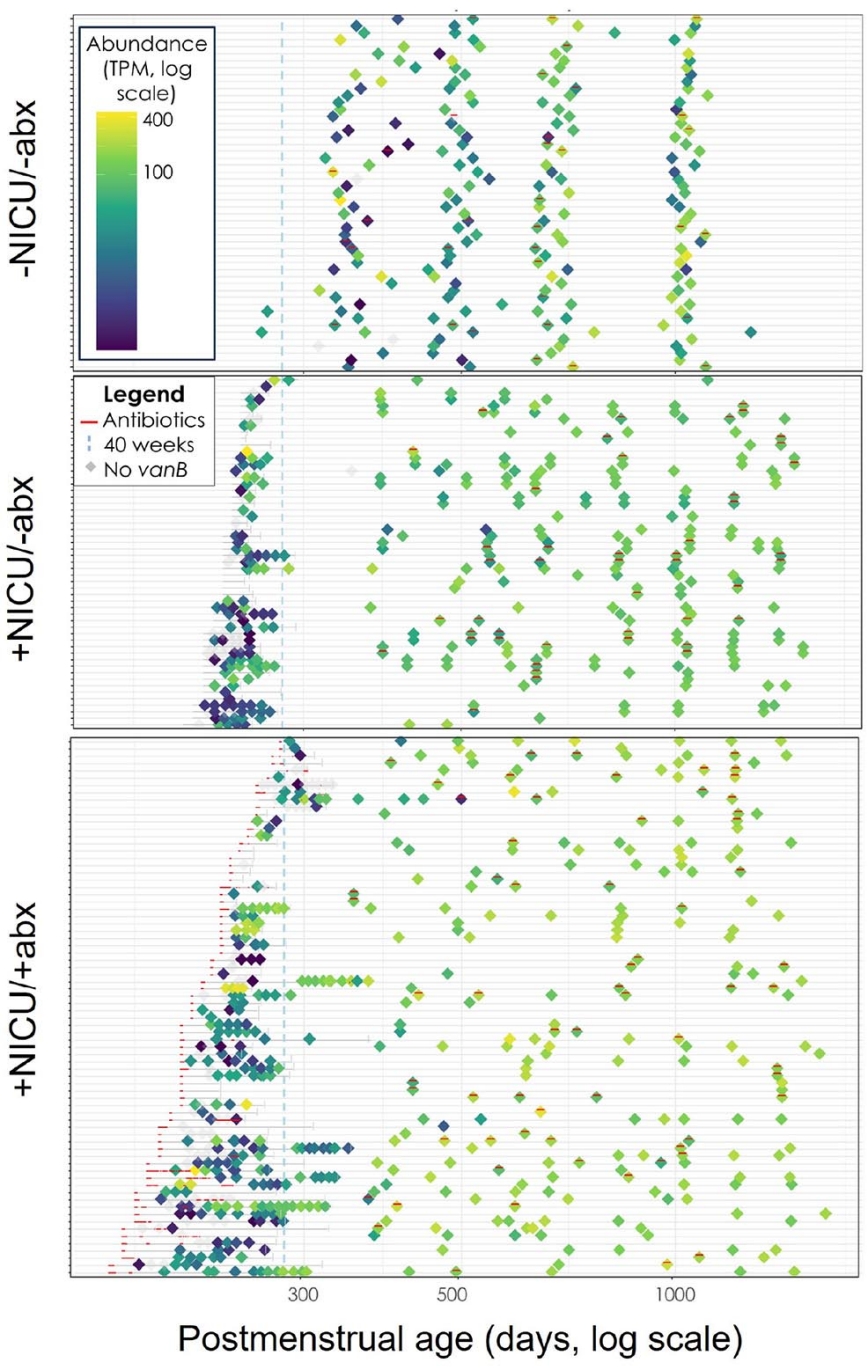
Heat Map of vanB Abundance Over Time A plot of each cohort is shown; each horizontal line represents one patient, with the patient’s stool samples represented by diamond shapes on that line. Abundance of vanB for each sample is shown on a color spectrum, with dark blue indicating the lowest abundance and bright yellow indicating the highest abundance. Antibiotic courses are shown with red lines (for post-discharge samples, antibiotics were given at any point in the prior 3 months). Samples with no vanB detected are shown in gray. The vertical dotted blue line represents 40 weeks postmenstrual age for reference.

**Results:**

The 3 study groups had significantly different resistomes longitudinally (Figure 2, p = 0.003). Some CI-ARG such as *vanB* were more abundant in children with any NICU stay, regardless of antibiotic use (Figure 3a, p = 0.0003). Unexpectedly, most CI-ARG were more abundant in the -NICU/-abx group at 3 years, including genes associated with resistance to carbapenems and cephalosporins (Figure 3b, p = 0.019; Figure 3c, p = 0.008, respectively). To improve visualization of longitudinal CI-ARG abundance at a patient level, we piloted a novel heat map, with an example of the map for *vanB* shown in Figure 4.

**Conclusion:**

This study showed long-term CI-ARG differences among children with and without initial NICU or antibiotic exposure. While *vanB* abundance increased in children exposed to antibiotics in the NICU, several CI-ARG were more abundant in the healthy infants. Next steps include analyzing taxonomic and clinical data to clarify the basis of these counterintuitive results.

**Disclosures:**

Rana F. Hamdy, MD, MPH, MSCE, FPIDS, StrepApp: "StrepApp"; patent application pending

